# Mindfulness-based Wellness and Resilience intervention among interdisciplinary primary care teams: a mixed-methods feasibility and acceptability trial

**DOI:** 10.1017/S1463423619000173

**Published:** 2019-07-01

**Authors:** Dana Dharmakaya Colgan, Michael Christopher, Sarah Bowen, Christiane Brems, Mathew Hunsinger, Brian Tucker, Eli Dapolonia

**Affiliations:** 1 Department of Neurology, Oregon Health & Science University, Portland, OR, USA; 2 School of Graduate Psychology, Pacific University, Hillsboro, OR, USA; 3 Virginia Garcia Memorial Health Center, Primary Care, Hillsboro, OR, USA

**Keywords:** resilience, mindfulness, primary care, burnout, feasibility, qualitative

## Abstract

**Aims:**

The primary objective of this study was to evaluate feasibility and acceptability of Mindfulness-based Wellness and Resilience (MBWR): a brief mindfulness-based intervention designed to enhance resilience and is delivered to interdisciplinary primary care teams.

**Background:**

Burnout is a pervasive, international problem affecting the healthcare workforce, characterized by emotional exhaustion, depersonalization, and decreased professional effectiveness. Delivery models of mindfulness-based resilience interventions that enhance feasibility for onsite delivery, consider cultural considerations specific to primary care, and utilize team processes that are integral to primary care are now needed.

**Methods:**

We conducted a mixed-methods feasibility and acceptability trial of MBWR. Primary feasibility and acceptability outcomes were assessed by number of participants recruited, percent of MBWR treatment completer, and attrition rate during the 8-week intervention, and four items on a Likert-type scale. Secondary outcomes of perceived effects were measured by focus groups, an online survey, and self-reported questionnaires, including the Brief Resilience Scale, the Five Facet Mindfulness Questionnaire-Short Form, and the Self-Compassion Scale-Short Form. Participants included 31 healthcare providers on interdisciplinary primary care teams employed a safety-net medical center. In the MBWR group, 68% identified as Latinx, compared to 64% in the control group.

**Findings:**

All criteria for feasibility were met and participants endorsed high levels of satisfaction and acceptability. The results of this study suggest that MBWR provides multiple perceived benefits to the individual healthcare provider, cohesion of the healthcare team, and enhanced patient care. MBWR may be a feasible and acceptable method to integrate mindfulness, resilience, and teamwork training into the primary care setting.

## Introduction

Burnout is a pervasive, international problem affecting the healthcare workforce, characterized by emotional exhaustion, depersonalization, and decreased professional effectiveness. In a 2017 survey of 14,000 physicians, 51% endorsed clinically elevated symptoms of burnout, reflecting a significant increase from 40% in 2013 (Peckham and Grisham, 2017). The highest rates of burnout reported are among emergency medicine and primary care providers (Shanafelt *et al*., 2015). Drivers of burnout include increased bureaucratic tasks, overall workload, poor life–work balance, lack of flexibility, autonomy, and control, misalignment of individual and organizational values, lack of social support/community at work, and loss of meaning in work (Balch *et al*., 2009; Shanafelt *et al*., 2012; Peckham and Grisham, 2017; West *et al*., 2018).

Personal, organizational, and societal consequences of burnout are significant. Physician burnout is associated with increased levels of anxiety, depression, and substance use. Female physicians commit suicide at about 2.3 times the rate of the general population and male physicians about 1.4 times (Shanafelt, 2009). Physical burnout also reduces patient access to care. Burnout is one of the strongest predictors of intent to reduce clinical work hours and leave current position (West *et al*., 2014), and nearly 20% of physicians reported an intent to reduce their clinical hours in the next year. The losses in patient services related to work cutback and early retirement have been estimated to be at least CAN$213 million (Dewa *et al*., 2014). Further, cross-sectional studies have linked physician burnout with suboptimal patient care practices (Williams *et al*., 2007, Klein *et al*., 2010), a doubled risk of medical error (Shanafelt *et al*., 2010), and a 17% increase in the odds of being named in a medical malpractice suit (Balch *et al*., 2011, West *et al*., 2018).

Combating burnout is a two-fold process that involves both individually focused and structural or organizational-directed solutions (West *et al*., 2018). Organizational-directed interventions that foster communication between members of the healthcare team and cultivate a sense of team cohesion and job control tend to be the most effective in reducing burnout (Regehr *et al*., 2014; Ruotsalainen *et al*., 2016; West *et al*., 2016; Panagioti *et al*., 2017). Individual approaches, such as mindfulness and resilience interventions, have been shown to decrease perceived stress, increase resilience to stressful work environments, and enhance work engagement (Ruotsalainen *et al*., 2016). Self-compassion is also pertinent to healthcare providers, as it is positively associated with resilience among medical residents and inversely associated with burnout among healthcare providers (Gilbert, 2010; Feldman and Kuyken, 2011; Hofmann *et al*., 2011; Olson *et al*., 2015). Self-compassion involves being touched by one’s own suffering, generating the desire to alleviate one’s suffering, and treating oneself with understanding and concern (Neff, 2003, Neff *et al*., 2005). Furthermore, increased self-compassion has been reported as a promising method of increasing resilience (Pidgeon *et al*., 2014).

Research must now address delivery models of mindfulness-based resilience trainings that enhance feasibility for onsite delivery, consider cultural considerations specific to primary care, and utilize team processes that are integral to primary care. Therefore, Mindfulness-based Wellness and Resilience (MBWR) was developed by the authors. MBWR was designed to be a brief, cost-effective, evidenced-based, and replicable curriculum to enhance mindfulness, resilience, and self-compassion among intact interdisciplinary primary care teams (IPCTs). MBWR is unique in that, it is delivered onsite among interdisciplinary teams with the aim to assist hospitals, medical centers, and training institutions in promoting health, well-being, and community among staff, ultimately, enhancing the quality of care they provide. The primary objectives of this trial were to (1) evaluate feasibility of recruitment and retention to a novel training program MBWR and (2) assess acceptability of MBWR training among IPCTs. The secondary objective was to determine the perceived effects of MBWR among IPCTs. To achieve these aims, we conducted a mixed-method, wait-list controlled trial.

## Method

### Participants

Recruitment and data collection occurred in a safety-net primary care center in the Pacific Northwest that serves predominately poor, uninsured, and underserved populations. As defined, primary care orientates toward family and community care and handles a wide array of patients and diseases states. Services include preventive care, physical examinations, and management of common, acute medical conditions. In addition, primary care provides care for chronic diseases and conditions, including diabetes, cardiovascular disease, mental health, and other long-term conditions. To be included in the study, individuals had to (1) be employed by the medical center; (2) be a member of an IPCT, including medical doctor, nurse, nurse practitioner, behavioral health consultant, physician assistant, medical assistant, or team assistant; (3) be willing to attend five of the eight sessions; (4) consent to complete baseline, post-, and 3-month follow-up MBWR measures; and (5) be fluent in English. Individuals were excluded if they endorsed active suicidality or psychosis, or attended a previous pilot study of MBWR. All participants provided written informed consent via a process approved by Institutional Review Board of Pacific University.

### Procedures

Two researchers attended primary care team meetings to inform 45 employees of the purpose of the study, the eligibility requirements, and exclusion criteria, and receive written informed consent from interested and eligible individuals. Researchers were experts in the fields of mindfulness and resilience for high stress populations. Expected recruitment was 80% of staff informed of the training at the medical center (*n* = 36). A battery of measures was collected on a secure web-based survey system and administered at three time points: baseline, immediately following the 8-week intervention period, and at 3-month follow-up. Following baseline assessments, IPCTs were assigned to either MBWR or waitlist control group (WL) in a 1:1 ratio. Due to the naturalistic study design, groups were allocated to treatment arm based on scheduling and clinic space availability. Participants were not blind to the groups. WL participants received the training after 3-month follow-up measures were complete.

### Intervention

MBWR, grounded in the evidence-based mindfulness practices of Mindfulness-Based Stress Reduction (Kabat-Zinn *et al*., 1985) and the Mindful Practice curriculum (Epstein *et al*., 2007), was developed by the authors and designed to increase resilience, mindfulness, and self-compassion among IPCTs. IPCTs typically consist of 7–14 members and include two to three physicians, and physician assistants, nurses and nurse practitioners, medical assistants, social workers, pharmacists, and community coordinators whom all work with the same panel of patients. Eight 60-minute weekly sessions were delivered onsite directly following weekly team meetings. Weekly sessions included “formal” mindfulness practices, or time set aside to engage in mindfulness practices such as body scan, mindful breathing, sitting meditation, loving-kindness, and mindful-movement. They also included “informal” mindfulness practices that are intended as way to intentionally apply the skills and qualities fostered in formal practice to daily living. Specific informal practices were developed for the primary care setting and used prior to entering an examination room, during patient–provider communication, professional consultation, or team meetings. For example, a provider may incorporate pausing before entering an examination room, to intentionally scan their body, breath, and mind states before walking into room. Class discussions explored how to integrate informal practices into the workday and create the structure and consistency needed to develop and maintain new skillful responses to stress and adversity in the workplace. Brief didactics on mindfulness, resilience, and relevant research were presented weekly. The primary interventionist was a doctoral student in clinical psychology and had extensive experience facilitating mindfulness-based interventions (MBIs) training in primary care settings. MBWR was provided to the active control group only. Following the completion of the 3-month follow-up assessments, the WL control groups received the intervention.

### Measures

#### Primary outcomes

**Feasibility**. Feasibility was assessed by number of participants recruited, percent of MBWR treatment completer, and attrition rate during the 8-week intervention. Recruitment of at least 80% of those screened and deemed eligible to participate was used to indicate feasibility. Similar to previous MBSR studies (Moss *et al*., 2014), treatment completer was defined as attending at least five out of eight sessions. An attrition rate equivalent or smaller than those reported in past MBI studies with healthcare providers (20%) was used to indicate MBWR feasibility (Shapiro *et al*., 2005).

**Acceptability**. Acceptability was measured by four items on a Likert-type scale (0–6): (1) How much did you enjoy this course? (2) How important was this course? (3) Would you recommend this course to colleague(s)? and (4) Would you participate in follow-up sessions?

### Secondary outcomes: perceived effects

#### Qualitative measures

Focus groups were conducted one week after the MBWR training to assess perceived effects of MBWR. The facilitator of the MBWR sessions conducted the two focus groups, one for each team that participated in MBWR. Approximately 7–10 questions were asked in each group (Table 1). Mindful inquiry, recognized as a valid qualitative interview process (Bentz and Shapiro, 1998), was employed to understand the participants’ first-person perspectives on how they experienced the training and its effects. Inquiry is a process in which a facilitator engages participants in a collaborative and interactive verbal exploration of their experiences and observations. This interview approach permitted discussion and allowed for data to enter the interview that was not directly sought, thus allowing participants to provide information they believe was important and relevant to them. Audio recordings of the focus groups were transcribed verbatim. To reduce the threat of social desirability bias, prior to the focus groups, electronic anonymous surveys with the same open-ended questions asked during the focus groups were sent to participants (Nederhof, 1985).

**Table 1.** Focus group questions

Questions	Follow-up questions
What was helpful about this class? What role does mindfulness play in your work day? How will you sustain the practice during the coming months/ years? How has taking this class together affected your team? How could this class be improved?	Can you describe how your response to stress has been affected because of this class? What did not work? What concerns do you have about this class?

**Quantitative measures:** The following self-report outcome measures were collected at baseline, post, and 3-month follow-up assessment points.

The Brief Resilience Scale (Smith *et al*., 2008) is a 6-item measure designed to assess the ability to bounce back or recover from stress. Higher scores indicate greater resilience. At baseline, the BRS demonstrated good internal consistency (*α* = 0.83).

The Five Facet Mindfulness Questionnaire-Short Form (Bohlmeijer *et al*., 2011) is a 25-item measure of dispositional or trait mindfulness based on the 39-item Five Facet Mindfulness Questionnaire (Baer *et al*., 2006). Higher scores for each facet indicate more of the trait. Due to previous reports of poor psychometrics, the Describing and Observing Facets were not assessed (Baer *et al*., 2008; Christopher *et al*., 2014). At baseline, the three facets of the FFMQ-SF (Acting with Awareness, Nonjudging of Inner Experience, and Nonreactivity to Inner Experience) demonstrated good-to-excellent internal consistency (*α*’s ranging from .75 to .91).

The Self-Compassion Scale-Short Form (SCS-SF) (Raes *et al*., 2011) is a 12-item measure that assesses three facets of self-compassion (self-kindness, mindfulness, common humanity) and their respective opposites (self-judgment, over-identification, isolation). Higher scores indicate greater self-compassion. At baseline, the SCS-SF demonstrated good internal consistency (*α* = .86).

### Sample size

As this was a feasibility study, a sample size calculation was not conducted. Instead, we followed the recommendations of Julious (2005), who suggested a minimum sample size of 12 subjects per treatment arm.

### Data analysis

#### Primary outcomes

Frequency reports analyzed feasibility and acceptability data and were performed using IBM SPSS version 22 (SPSS, 2013).

### Secondary outcomes

#### Qualitative

Prior to analysis, focus group transcripts were de-identified to ensure confidentiality and limit analytical bias among researchers. Qualitative data were analyzed using a conventional content analysis. This method systematically examines material and obtains a condensed description of content (Hsieh and Shannon, 2005). The first author independently reviewed the focus group transcripts in their entirety to get an overall sense of the data. Next, each transcript was individually re-read to identify recurring words, phrases, or concepts and the first author and a research associate independently developed preliminary codes (open coding). The two researchers discussed their independently developed codes, resolved differences, and devised a final coding scheme. The final coding scheme was then applied to both the transcripts by the two independent coders. Once all transcripts had been coded, the first author examined all data within a particular code. Codes were then sorted into categories based on how different codes are related and linked. These emergent categories were used to organize and group codes into meaningful cluster. Some codes were combined during this process, whereas others were split into subcategories. Definitions for categories were developed (Coffey and Atkinson, 1996, Hsieh and Shannon, 2005, Patton, 2005). Transcripts were then reanalyzed to search for disconfirming data. A similar, yet independent, process was completed for the online surveys.

#### Quantitative

Means and standard deviation were calculated for each variable (resilience, mindfulness, and self-compassion) at the three time points. Analyses of between-group effects of mindfulness, resilience, and self-compassion were tested individually using a multilevel linear modeling (MLM) approach with restricted maximum likelihood estimation (REML), performed using IBM SPSS version 22 (SPSS, 2013). Statistical significance for all parameter estimates were set at *p* < .05, two tailed. Effect sizes were calculated using Cohen’s *d* (Cohen, 1992).

## Results

### Preliminary analyses: feasibility and acceptability

Of 45 primary care team members screened, six individuals did not meet study eligibility because they were unable to attend at least five of the eight classes. This was due to maternity leave (*n* = 1), scheduling conflicts due to clinical rotations (*n* = 4), or pending resignation (*n* = 1). One individual declined to participate due to a religious conflict with mindfulness meditation. Thirty-eight individuals enrolled in the study and completed baseline assessments (84%). Due to unexpected structural changes in the clinic, at the beginning of the study, seven participants (six medical assistants and one registered nurse) were required to switch teams. To reduce the threat of contamination, their data were removed. The eligible study sample numbered 31 participants. Two teams (*n* = 9, *n* = 7) completed MBWR and two teams were in a WL control (*n* = 7, *n* = 8). Of the 31 participants, 84% identified as female; 71% identified as Mexican, Latinx, or Puerto Rican, 20% as White, 6% as Asian, and 3% as Black. Medical assistants comprised 29% of the sample, primary care physicians comprised 23%, nurse or nurse practitioners 23%, team assistants 6%, physician’s assistants 3%, resident pharmacists 3%, social workers 3%, and other 10% (community resource officers, interns; see Table 2).

**Table 2.** Demographics and professional roles by group

	Treatment group		
Demographic variables	MBWR (n = 16)	Wait list (n = 15)	Total
Gender	87% women (14)	80% women (12)	26 women (84%)
Ethnicity	69% Latinx (11)	74% Latinx (11)	71% Latinx (22)
	25% White (4)	20% White (3)	23% White (7)
	6% Asian (1)	7% Black (1)	3% Asian (1)
			3% Black (1)
Professional role	25% Medical assistants (4) 13% Physicians (2) 13% Nurse, nurse practitioners (2) 6% Physicians assistants (1) 6% Clinical pharmacist (1) 12% Team assistance (2) 6% Social workers (1) 19% Others (3)	33% Medical assistants (5) 33% Physicians (5) 20% Nurse, nurse practitioners (3) 14% Others (2)	29% Medical assistants (9) 23% Physicians (7) 16% Nurse, nurse practitioners (5) 3% Physicians assistants (1) 3% Clinical pharmacist (1) 6% Team assistance (2) 3% Social workers (1) 16% Others (5)

All MBWR participants were completers (ie, attended at least five out of eight sessions) and total class attendance was 88%. Six participants attended all eight sessions, eight participants attended seven sessions, one participant attended six sessions, and two participants attended five sessions. Reasons for missing a class included being off-shift, attending an off-site training, or responding to a medical crisis or labor and delivery. Online surveys revealed participant ratings of the MBWR course: 87% of participants reported *extremely* or *very much* enjoying the course, 82% rated the course as *extremely* or *very important*, 100% would recommend the course to a colleague, 100% reported they would attend follow-up or booster sessions, and 100% reported the instructor was *extremely* or *very knowledgeable*.

### Secondary outcome: perceived effects

#### Qualitative

Analysis of the focus groups and open-ended survey questions from MBWR participants revealed seven themes: (1) increased nonreactive awareness, (2) improved adaptive coping, (3) enhanced team cohesion, (4) enhanced quality of patient–provider communication, (5) increased quality of life, (6) participants’ perceived importance of integrating informal mindfulness practices into the workday; and (7) participants’ recommendations for longer and more frequent sessions. Each of the themes and subthemes, with illustrative participant quotes, are displayed in Table 3.

**Table 3.** Final qualitative coding scheme

Increased nonreactive awareness	I am much more aware of my emotions. I recognize my stress sooner. It was helpful [to develop] the awareness of how we tend to react, either positively or negatively, to a stimulus, and learning to stop and be aware of the reaction instead of judging the reaction.
Increased adaptive coping	I have learned to do a very quick re-centering which I can use in the middle of a very busy day to get back to balance quickly. [It has helped] being able to take a simple moment to step back and regroup when we have an emotionally difficult situation with a patient or each other.
Enhanced team cohesion	If just feels better, and cohesive. Kind of like of knowing that we are in this together and we can do this. Like team spirit. There is more “team-ness”.
Communication	[The training] has helped us to have better communication with each other, listen to each other, and work as a team.
Social support	We have more trust among each other. I am more comfortable with my teammates.
Common humanity	It is helpful to know that you are not the only one dealing with stressful situations.
Increased quality of patient–provider communication	I can focus my attention better and longer when [I am] with patients and colleagues.
Enhanced quality of life	Even at home. It has been helpful to use [practices] in situations that maybe are not necessarily stressful but in situation where perhaps in the past I Would not have paid as much attention and I feel that am appreciate some of those activities maybe more than I would have before.
Integration into the work day	Walking for 10 minutes at the end of my lunch break every day has been wonderful, but it has also made me more aware of other mindfulness activities I can slip in on an ongoing basis.
	I like the star on the door. That star I saw every time I went into a room. Something that I see all the time [was helpful]. I’ve really enjoyed adding mindfulness to our team meetings and also knowing I can count on my team members to remind me to be mindful.
Recommendations to improve training	Course could be longer both in terms of number of weeks and minutes per session. “Booster" classes periodically would be great. More Frequent (more sessions per week)

#### Quantitative

Due to the nature of the study and the small sample size, analyses focused on direction and magnitude of mean change from baseline to post-course in variables of resilience, mindfulness, and self-compassion, although results of signiﬁcant tests are also provided (Table 4). To assess intervention effects, we estimated MLM separately for each outcome variable, using REML. Past meditation experience and expectancy of treatment effectiveness were included as covariates in the models. At baseline, there were significant differences between MBWR and WL in resilience (*P* = .006) and mindfulness non-reactivity of internal experience (*P* = .02). To account for these differences, for each of these dependent variables, the pre-MBWR variable was entered into the respective model as a covariate, and MLM analyses of co-variance were performed (Tabachnick and Fidell, 2007).

**Table 4.** Means, standard deviations, and change in outcome variables for MBWR and wait-list control groups at baseline, post-MBWR, and 3-month follow-up

Outcome variables	MBWR			Control		
	Pre	Post	3FU	Pre	Post	3FU
	M (SD)	p	p			
BRS	18.23 (2.77)	23.06 (2.08)	24.14 (2.48)	15.64 (2.67)	19.21 (2.75)	18.10 (2.88)
Total FFMQ	51.76 (6.56)	51.86 (7.32)	55.36 (8.55)	49.50 (6.44)	48.08 (6.11)	49.50 (5.83)
NR	17.23 (3.03)	17.86 (3.39)	18.57 (4.13)	14.35 (2.95)	13.16 (2.55)	14.40 (2.98)
AWA	18.30 (3.67)	17.40 (3.09)	18.71 (3.45)	18.50 (3.52)	18.66 (2.74)	18.80 (3.52)
NJ	16.23 (4.66)	16.60 (4.47)	18.07 (4.29)	16.64 (2.95)	16.20 (3.74)	16.30 (3.68)
SCS	42.64 (8.20)	45.46 (7.13)	46.13 (8.48)	37.71 (7.09)	36.66 (8.70)	38.20 (7.13)

Note: 3FU = 3-month follow-up; BRS = Brief Resilience Scale; Total FFMQ = Three Facets (Act with Awareness, Non-Judgmental Awareness, and Nonreactivity) of Five Facets Mindfulness Questionnaire; NR = Nonreactivity of Inner Experiences Facet of FFMQ; AWA = Act with Awareness Facet of the FFMQ; NJ = Nonjudgmental Awareness Facet of the FFMQ; SCS = Self Compassion Scale – Short Form.

## Discussion

MBWR was developed by the authors to be a brief, cost effective, evidenced-based, and replicable curriculum that is delivered onsite for IPCTs. MBWR is designed to assist hospitals, medical centers, and training institutions in promoting health, well-being, and community among staff, ultimately, enhancing the quality of care they provide. The primary aim of this study was to assess feasibility and acceptability of novel intervention, MBWR. All criteria for recruitment and retention were met and participants endorsed high levels of satisfaction and recommendations to colleagues. Results indicate that MBWR may be a feasible and acceptable method to integrate mindfulness and resilience into the primary care setting. This study offers several unique contributions to the literature. First, to our knowledge, this is the first study to examine a mindfulness-based intervention enriched with resilience that is delivered to IPCTs in the natural workday environment. Second, MBWR was implemented in a safety-net medical center, designed to reduce health disparities that disproportionately affect racial and ethnic minority groups, and poor and uninsured individuals. These centers report higher rates of burnout and turnover among healthcare workers than in non-safety-net center (Werner *et al*., 2008). Further, MBWR participants reported increased quality of care, described as enhanced focus and less reactivity when interacting with patients. These preliminary findings suggest that MBWR training for providers may have secondary benefits for the marginalized communities they serve. Finally, 68% of the sample identify as Latinx, a population greatly underrepresented in the mindfulness and resilience literature. Results suggest that MBWR was feasible and acceptable for these participants of Latinx heritage.

The study also revealed a potential limitation of this delivery model, as seven participants were required to switch teams during the study, moving from the control group to the intervention group (or vice versa). This unforeseen reduction in sample size may have reduced the ability to see true differences between the groups. Researchers will need to engage in careful consideration and thoughtful planning when developing future studies using this delivery method in the primary care setting as to reduce threat of contamination.

Secondary objectives were to determine the perceived effects of MBWR among medical providers who attended the training. Qualitative analyses of focus groups and online survey were conducted to achieve this aim. Participants of MBWR described (1) enhanced self-awareness, (2) increased self-regulation skills, and (3) increased team cohesion, congruent with the three essential aims of resilience-promoting programs (Epstein and Krasner, 2013). Existing literature suggests that to enhance resilience in the face of stressful work conditions, medical providers must be able to recognize when they are adversely affected by stress, cultivate skillful responses to the stressors, and self-regulate their cognitive, emotional, somatic, and behavioral reactions to the stressors (Shapiro *et al*., 2005; Wolever *et al*., 2012; Epstein and Krasner, 2013; Schroeder *et al*., 2016; Colgan *et al*., 2018; West *et al*., 2018). Following MBWR, participants reported increased awareness and non-reactivity of inner thoughts, emotions, and bodily sensations. Further, this increased awareness may have afforded an expanded behavioral repertoire and influenced participants’ reported increase in adaptive coping to stress or adverse conditions, permission and time devoted to personal growth.

The participants also described enhanced team cohesion and a greater sense of community, following the training. Three elements of team cohesion were revealed as improved communication, increased social support, and a greater sense of shared common humanity among teammates. This is a noteworthy finding because previous research has shown that members of highly cohesive teams are more likely to contribute equally to problem solving, are not as likely to be adversely affected by the power and status structures within the groups (Secord and Backman, 1964), and contribute to increased provider satisfaction, which effectively predicts turnover (Lucas *et al*., 1993; Tumulty *et al*., 1994; Leveck and Jones, 1996; Wells *et al*., 2002) and reduces burnout among healthcare providers (Lasalvia *et al*., 2009). Implementing MBWR in primary care teams may be an effective mechanism to facilitate enhanced community.

Additionally, MBWR participants emphasized the importance of integrating informal mindfulness practices into the workday. Fundamental to these efforts is the creation of a “container” of deliberate and consistent practice within which the culture of mindfulness can be cultivated and sustained. Humans become skilled at what they habitually do (Epstein, 2017); therefore, providing opportunities for IPCTs to train together and create tailored, authentic workflows that incorporate informal mindfulness practices may reduce sympathetic nervous system activation, improve emotion regulation, and enhance coping with psychological challenges (Hölzel *et al*., 2013; Duchemin *et al*., 2015; Westphal *et al*., 2015). Brief, yet frequent, informal mindfulness practices within this population may bolster individual mental immunity, as well as develop and sustain a culture of mindfulness-based resilience within the primary care work environment. The strong emphasis on informal practices may also reflect a greater sense of collectivism, congruent with the Latinx culture (García-Campayo *et al*., 2017).

Focus groups and the online survey inquired about negative reactions or concerns regarding the training. The most frequent concern was the length: Participants requested that the training be longer (duration of training) and more frequent (more than one day a week). No other concerns or negative reactions were noted.

The results from this study must be interpreted with caution. Limited funding and the nature of the study design afforded several limitations. The small sample size reduced generalizability of the findings. Individual interviews were not conducted and the interventionist conducted the focus groups. As a result, social desirability bias may have influenced participants’ responses during the focus groups. Additionally, the interventionist was one of the researchers who analyzed the qualitative data. Further, group composition may have biased the findings. Future larger clinical trials are needed to explore the effects of MBWR on providers’ health, perceived burnout, patient–provider communication and relationship, while also exploring potential mechanisms of MBWR.

It is in healthcare institutions’ best interest to support the effort of all members of the workforce to enhance their capacity for resilience (Krasner *et al*., 2009; Linzer *et al*., 2014). The unique delivery model of MBWR, provided in the medical setting during paid-protected time and delivered to intact primary care teams, reflects the healthcare institution’s intention to support the workforce. The results of this study suggest that MBWR may assist in the cultivation and sustainability a thriving and flourishing primary care community and illustrate the potential benefits of an institutional commitment to provider well-being, that may offer at least a partial solution to the current crisis of physician burnout.
